# Optimizing the management of congenital thrombotic thrombocytopenic purpura

**DOI:** 10.1016/j.rpth.2025.103270

**Published:** 2026-01-20

**Authors:** Melissa F. Glasner, Senthil Sukumar, Spero R. Cataland, Marie Scully, Shruti Chaturvedi

**Affiliations:** 1 Department of Medical Education, The France Foundation, Old Lyme, Connecticut, USA; 2 Hematology & Oncology, Baylor College of Medicine, Houston, Texas, USA; 3 Division of Hematology, Richard J. Solove Research Institute, The Arthur G. James Cancer Hospital, The Ohio State University, Columbus, Ohio, USA; 4 Haemostasis and Thrombosis, Hematopathology and Blood Transfusion, University College London Hospitals, London, United Kingdom; 5 Division of Hematology, Johns Hopkins University, Baltimore, Maryland, USA

**Keywords:** ADAMTS-13 deficiency, anemia, congenital TTP (cTTP), multidisciplinary, plasma therapy, platelet disorder, recombinant ADAMTS-13, thrombocytopenia, thrombotic microangiopathy

## Abstract

**Background:**

Congenital thrombotic thrombocytopenic purpura (cTTP) is a rare, life-threatening thrombotic microangiopathy caused by genetic mutations in the *ADAMTS-13* gene, leading to severe enzyme deficiency. This results in the accumulation of ultralarge von Willebrand factor multimers, which trigger platelet aggregation, microangiopathic hemolytic anemia, thrombocytopenia, and organ damage. cTTP episodes are often triggered by physiological stressors and have a bimodal age of presentation.

**Objectives:**

To summarize the clinical course, complications, and advances in treatment of cTTP, highlighting the role of recombinant ADAMTS-13 (rADAMTS-13).

**Methods:**

A review of registry data, clinical studies, and expert guidelines was conducted to assess cTTP pathophysiology, presentation, and therapeutic approaches.

**Results:**

Acute cTTP episodes manifest variably, often requiring a “second hit,” such as infection or pregnancy, for disease induction. Chronic complications include cardiovascular disease, chronic kidney disease, and neurocognitive impairments, contributing to increased morbidity and mortality. Plasma infusions are effective but are associated with logistical challenges and adverse events. rADAMTS-13, a novel therapy approved in 2023, demonstrated superior efficacy in preventing acute episodes and reducing treatment burden, with fewer adverse events compared with standard plasma therapy.

**Conclusion:**

cTTP management necessitates individualized care by a multidisciplinary team. Treatment of cTTP relies on ADAMTS-13 replacement, which may be more effectively delivered with rADAMTS-13 than with plasma infusions. Further studies are needed to evaluate its long-term safety, particularly during pregnancy, and to optimize treatment strategies.

## Essentials

• Congenital TTP (cTTP) is a rare, inherited disorder caused by biallelic ADAMTS13 mutations, leading to recurrent microvascular thrombosis, hemolytic anemia, and organ damage.
• This review draws on international hereditary TTP registries, clinical studies of plasma and recombinant ADAMTS13 therapy, and real-world patient experiences, including pregnancy management.
• Plasma therapy reduces acute episodes but is burdensome and inconsistently effective, while recombinant ADAMTS13 shows improved prophylactic efficacy, safety, and convenience.
• rADAMTS13 represents a major advancement in cTTP management, offering a targeted, potentially safer, and more sustainable treatment option that may improve long-term patient outcomes.

## 1 Continuing Medical Education Syllabus

### 1.1 Overview of thrombotic thrombocytopenic purpura

Thrombotic thrombocytopenic purpura (TTP) is a life-threatening thrombotic microangiopathy characterized by widely disseminated microvascular thrombi in the terminal arterioles and capillaries [1]. TTP is driven by a severe deficiency of ADAMTS-13, the metalloproteinase principally responsible for cleaving von Willebrand factor (VWF) multimers into physiologic sizes [2]. When ultralarge VWF multimers accumulate, aberrant adhesion and aggregation of platelets result in microangiopathic hemolytic anemia, thrombocytopenia, and end-organ damage [1].

For the most common form of TTP, immune-mediated TTP (iTTP), ADAMTS-13 deficiency is caused by anti–ADAMTS-13 autoantibodies that functionally inhibit or enhance clearance of ADAMTS-13 [3]. More rarely, ADAMTS-13 deficiency can be hereditary in individuals with biallelic mutations in the *ADAMTS-13* gene, which is known as congenital TTP (cTTP) [1]. This is an ultrarare disorder, with an annual incidence of <1 per million, and comprises only 5% of all cases of TTP [4]. Certain populations have a higher prevalence of cTTP, namely neonates and children, though notably up to 25% of women diagnosed with TTP during their first pregnancy have cTTP as opposed to iTTP [5,6].

Acute cTTP episodes are characterized by microangiopathic hemolytic anemia, thrombocytopenia, and end-organ damage [7]. Severe ADAMTS-13 deficiency alone is not always sufficient to induce the clinical syndrome, which commonly requires an additional physiologic stressor, or “second hit” [8]. As a result, exacerbations of cTTP commonly occur during periods of increased VWF expression, such as the neonatal period, pregnancy, or acute infection or inflammation [9].

In contrast to iTTP, exacerbations of cTTP may present with a milder degree of hemolysis and/or thrombocytopenia [9]. The incidence of acute kidney injury is also more common in cTTP than in iTTP [10]. Some patients may experience neurological events from cTTP in the absence of thrombocytopenia, and others may have laboratory evidence of an exacerbation yet remain asymptomatic [9]. The annual incidence of acute cTTP episodes over lifetime is 0.35 per person-year [9], with a bimodal distribution across age of presentation.

During childhood, the median age of diagnosis is 3 years; in young adulthood, during first pregnancy, the median age of diagnosis is 31 years [1,11]. Prespacer domain mutations in *ADAMTS-13* are associated with an earlier age of onset compared with postspacer domain mutations (24 months vs 294 months, *P* < .0001) [6]. The highest incidence of acute episodes occurs in those aged <10 years, with decreasing incidence in older patient groups [9]. Younger patients may also exhibit more severe end-organ damage [9].

### 1.2 Chronic impact of cTTP

**Figure 1:**
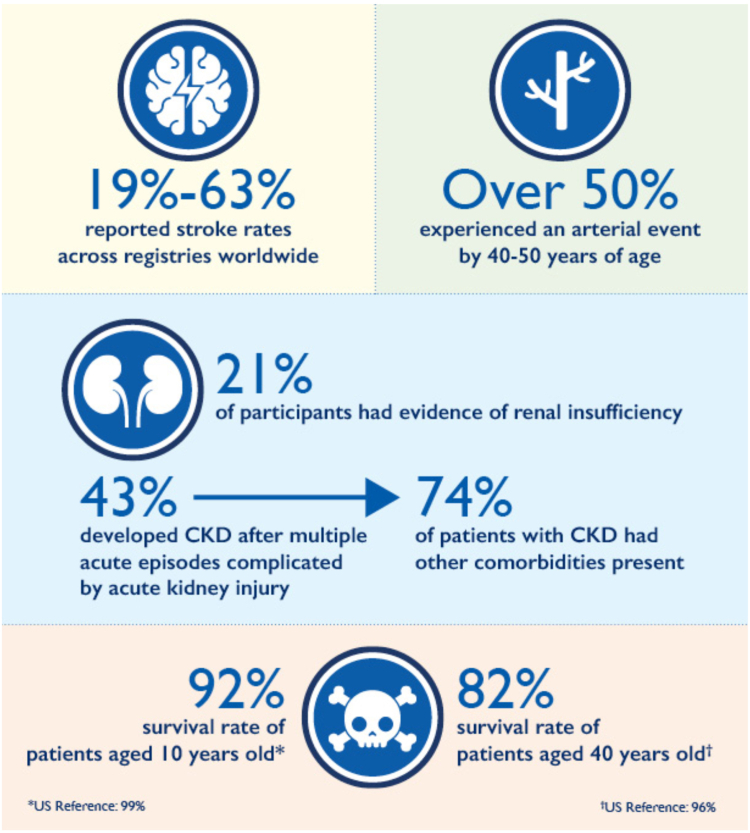
Clinical outcomes of congenital thrombotic thrombocytopenic purpura. Patients with congenital thrombotic thrombocytopenic purpura show high rates of stroke, arterial events, renal disease, and reduced survival compared with population references. CKD, chronic kidney disease.

In addition to acute exacerbations, those affected by cTTP also experience chronic complications (Figure 1). In a report from the UK Hereditary TTP Registry, the leading causes of initiating prophylactic therapy were persistent thrombocytopenia, headache or migraine, lethargy, strokes and transient ischemic attacks, abdominal pain, and recurrent exacerbations with microangiopathic hemolytic anemia [6]. The burden of these complications often leads to long-term end-organ damage, neurocognitive impairment, depression, and increased mortality [7,12].

Cardiovascular disease, particularly stroke and myocardial infarction, is prevalent in patients with cTTP, often occurring at younger-than-expected age [13]. Stroke events occur more frequently than myocardial infarction, with reported stroke rates of 19% to 63% across registries worldwide [6,7,13]. In some studies, >50% of cTTP patients experienced an arterial event by age 40 to 50 years [6,7]. Reduced ADAMTS-13 activity, which is thought to be a risk factor for the development of cardiovascular disease and stroke in patients with iTTP [14], may also play a role in these cTTP outcomes. However, there is conflicting evidence in the literature.

Chronic kidney disease (CKD) is also common in cTTP. In the International Hereditary TTP Registry, 21% of participants had evidence of renal insufficiency, with 43% developing CKD after multiple acute episodes complicated by acute kidney injury [9]. Of those with CKD, 74% also had other comorbidities present, though there was no significant correlation between CKD onset and other comorbidities [9].

cTTP is also associated with increased mortality compared with a US reference population. Patients aged 10 years had a 92% survival rate compared with a US reference of 99%. This gap further widens over time, with patients aged 40 years having an 82% survival rate relative to the national baseline of 96% [7].

### 1.3 Treatment approaches in cTTP

Patients with cTTP require vigilant monitoring, especially throughout pregnancy. Regular clinical and laboratory assessment is essential, focusing on platelet counts, hemolytic markers, and ADAMTS-13 activity levels [15]. The mainstay of therapy is enzyme replacement, typically with fresh-frozen plasma (FFP) infusions or, where available, recombinant ADAMTS-13 (rADAMTS-13), administered either prophylactically or in response to symptoms to maintain adequate ADAMTS-13 levels. In some regions, clinicians have successfully used plasma-derived VWF-factor (F)VIII concentrates, which also contain ADAMTS-13, to treat patients with cTTP [16,17]. VWF-FVIII products, such as Koate and Alphanate, have been found to contain relatively high levels of ADAMTS-13 activity, and clinical improvements have been reported following their use in cTTP patients [17]. However, there is currently no clinical indication for the use of these agents in patients with cTTP.

### 1.4 Plasma infusions

Transfusions of FFP can be used for both the prevention and treatment of acute cTTP episodes [18]. Plasma infusions work by providing sufficient quantities of ADAMTS-13 from donor plasma. In more severe cases, plasma exchange has also been utilized. For acute on-demand therapy, the International Society on Thrombosis and Haemostasis (ISTH) Good Practice Statement recommends plasma infusions (10-15 mL/kg body weight) daily until improvement (ie, platelets normalize and symptoms resolve) [18]. FFP is most frequently used, with a median time to response of 4.0 days (range, 0.0-43 days) [9].

The optimal level of plasma ADAMTS-13 activity to maintain platelet count and manage symptoms is currently unknown. Clinicians determine the need for prophylaxis to prevent acute exacerbations on a case-by-case basis, guided by the patient’s chronic symptoms, exacerbation frequency, and ability to receive or tolerate treatment. The most common prescription is 10 to 15 mL/kg every 2 to 3 weeks, which is typically sufficient to control clinical and laboratory manifestations while being convenient and resource efficient [19]. However, there is limited high-quality clinical evidence supporting or refuting this regimen. Alternative approaches include episodic or on-demand infusions around known triggers.

Plasma infusion prophylaxis has shown mixed efficacy results to date. In the International Hereditary TTP Registry, 87 patients were followed for a median of 4.2 years (range, 0.01-15), with 43 receiving regular plasma prophylaxis [9]. No significant difference was observed in the annual incidence of acute TTP episodes between those on plasma prophylaxis (0.36 episodes/y; 95% CI, 0.29-0.44) and those without prophylaxis (0.41 episodes/y; 95% CI, 0.30-0.56) [10]. In the UK TTP Registry, 49 of 73 patients were on regular prophylaxis, with 23% starting due to thrombocytopenia and 66% due to symptoms [6]. Prophylaxis significantly reduced symptoms, including a reduction in cerebrovascular accidents (2% vs 17%; *P* = .04) [6,8].

Additionally, maintaining a plasma prophylaxis schedule can be challenging for many patients. While 50% to 70% of patients with cTTP are on regular plasma prophylaxis, approximately 30% of patients with cTTP-related major morbidities do not receive prophylaxis. A dosing frequency of every 2 to 3 weeks is achieved in 2 of 3 patients, regardless of the dose, while 80% of patients on prophylaxis receive at least 15 mL/kg every 2 weeks [6,9,20].

There are several complications associated with plasma transfusion, including the rare risk of developing ADAMTS-13 alloantibodies, transfusion-transmitted infections, and transfusion reactions [21]. In a US cohort of cTTP patients, 21 of 25 (84%) experienced allergic reactions, 2 of which were anaphylactic [22]. In patients with cTTP who are in remission, the guidelines suggest either prophylaxis or a watch-and-wait strategy [18].

There are several other factors when considering prophylactic plasma transfusions for patients. Receiving treatment at a hospital or infusion clinic often requires several hours to complete, leading to time away from work, school, or social activities [20]. The associated costs, including travel and treatment, can add a significant burden, further contributing to inconvenience and negatively impacting quality of life (QoL) [20].

Additionally, intravenous access is essential for these treatments, which means repeated venipunctures that can cause scarring and loss of peripheral access over time. In some cases, ports or central lines are used; however, they carry a risk of complications, such as thrombosis or infection, adding to the overall challenge of managing these treatments (Supplementary Video 1).

### 1.5 rADAMTS-13

The United States Food and Drug Administration approval of rADAMTS-13 in late 2023 represents a major advancement in the treatment of cTTP [23]. rADAMTS-13 allows for the targeted replacement of ADAMTS-13 deficiency in cTTP [24]. This therapy can be utilized for both prophylaxis and treatment of acute cTTP episodes. rADAMTS-13 is produced without any human or animal additives, making it a purified therapeutic option available to those who cannot tolerate or will not accept plasma products (eg, in the setting of prior anaphylaxis or religious beliefs such as the Jehovah’s Witness faith).

In a phase 3, open-label, crossover trial, 48 patients were randomly assigned to receive either rADAMTS-13 or standard therapy (plasma infusion or intermediate FVIII product) in two 6-month periods, followed by an additional 6 months of rADAMTS-13 for all [24]. The primary efficacy outcome was the occurrence of acute TTP events. Results showed that no acute TTP events occurred during prophylaxis with rADAMTS-13, while 1 acute event occurred with standard therapy [24]. Thrombocytopenia was also less frequent with rADAMTS-13 compared with standard therapy [24].

rADAMTS-13 has a safer profile with fewer adverse events compared with standard therapy (71% vs 84%) and a lower rate of drug-related adverse events (9% vs 48%) [24]. Most adverse events were mild to moderate, with the most common event being headache (31.3%). Additionally, there were no reported hypersensitivity reactions during rADAMTS-13 therapy, compared with 16 patients who experienced hypersensitivity reactions with standard therapy [24]. No neutralizing antibodies developed during rADAMTS-13 therapy. The mean duration of measurable ADAMTS-13 activity (>10%) was also substantially longer for rADAMTS-13 at 5.2 days (95% CI, 4.9-5.5) vs standard therapy at 1.7 days (95% CI, 1.2-2.2) [24]. rADAMTS-13 administration also resulted in higher peak ADAMTS-13 activity levels compared with standard therapy.

Currently, the recommended dosing for prophylaxis is 40 U/kg every other week or weekly. On-demand (acute) therapy is dosed once daily: 40 U/kg on day 1, 20 U/kg on day 2, and 15 U/kg on day 3 onward, until resolution of clinical symptoms and normalization of platelet count. rADAMTS-13 also decreases the time toxicity of treatment, as it is administered as a small-volume intravenous bolus, which is convenient for home-based therapy. This represents a significant shift in the current management of cTTP, providing patients with a less burdensome and more sustainable treatment option that has greater efficacy than the current standard of care. For patients with cTTP, the updated 2025 ISTH guidelines support ADAMTS-13 replacement over FFP, when accessible. Otherwise, FFP may still be effective (Supplementary Video 2) [25].

### 1.6 cTTP and pregnancy

Patient monitoring should be intensified during the third trimester and postpartum period, as episodes can occur during these times [15,26]. Early recognition and differentiation from other pregnancy-associated thrombotic microangiopathies, such as preeclampsia or hemolysis, elevated liver enzymes and low platelets (HELLP) syndrome, are critical due to overlapping clinical features and high maternal and fetal morbidity with delayed therapy [27]. Aspirin may be considered in women with additional risk factors, such as a history of preeclampsia, antiphospholipid syndrome, or other indications for thromboprophylaxis [28,29].

There is limited high-quality evidence to assess the efficacy of plasma therapy during pregnancy [20]. Pregnancy can trigger cTTP exacerbations when ADAMTS-13 activity falls <10%, often leading to fetal loss and poor maternal outcomes [20]. Plasma infusions may help improve outcomes, with a recommended dosage of 10 to 15 mL/kg every 2 weeks during pregnancy, though this may need to be increased to weekly or twice-weekly on a case-by-case basis. Close monitoring by an expert center is advised throughout pregnancy [18]. The ISTH evidence-based guidelines recommend prophylactic treatment over no prophylactic treatment in patients with cTTP who are pregnant [18].

The safety of rADAMTS-13 in pregnancy has not been established. Clinicians must weigh the potential benefits against the risks on an individual basis, using shared decision-making. In a long-term extension study, 2 patients became pregnant while on rADAMTS-13 prophylaxis and were subsequently discontinued from the trial [23,24]. One experienced a miscarriage, which was deemed unrelated to the therapy, while the other delivered a healthy baby [24]. Under a compassionate use program, 2 additional cTTP patients received rADAMTS-13 during pregnancy. Both delivered healthy babies, with 1 patient achieving clinical remission and delivering at 29 weeks without adverse effects linked to the therapy [24].

Recently, the successful use of rADAMTS-13 as rescue therapy for an acute exacerbation of cTTP during the third trimester of pregnancy has been reported [27]. Due to the severity of her initial presentation, the patient was treated with rADAMTS-13 40 U/kg daily until platelet count was > 100 × 10^9^/L, with subsequent induction of labor, followed by standard dosing protocol. After successful delivery of a healthy infant, the patient was placed on prophylaxis therapy with rADAMTS-13 and continues to do well [27,30]. In a recently published case study, rADAMTS-13 was well-tolerated in a pregnant woman, with no symptom flare-ups or development of anti–ADAMTS-13 antibodies.

While rADAMTS-13 may offer a safe and effective alternative to plasma infusions for preventing cTTP complications in pregnancy, further research is needed to determine the optimal dosing regimen.

### 1.7 Patient considerations for therapy

In a study examining the impact of cTTP, patient interviews (11 adults) revealed that fatigue, headaches, bruising, and joint pain were the most common and disturbing symptoms of cTTP [31]. The interviews emphasized the delayed diagnoses, continuous treatment regimens, and the burdens of both symptoms and treatments. The impact of cTTP was categorized into 5 main areas: emotional, activity restrictions, work/study limitations, bleeding, and other physical effects. Emotional impacts, such as anxiety, depression, mood swings, and financial distress, were the most frequently reported and most disturbing [31]. Work/study restrictions were commonly noted but were less disturbing overall.

Patients also emphasized the need for safer and more convenient treatment options [30]. This study provides a more patient-centered view of cTTP burden, emphasizing the need for disease-specific tools to assess QoL and treatment outcomes, as generic instruments do not capture the full spectrum of the disease’s effects [30].

**Figure 2:**
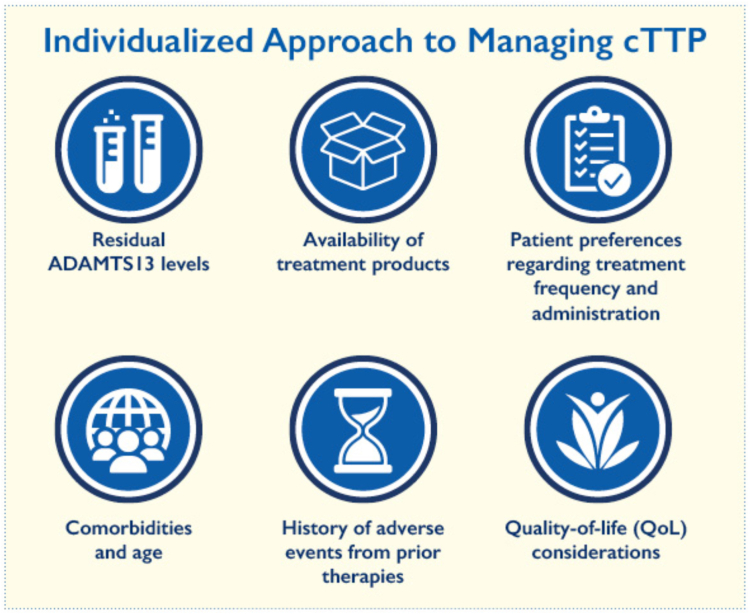
Individualized approach to managing congenital thrombotic thrombocytopenic purpura (cTTP). Treatment decisions incorporate residual ADAMTS-13 activity, product availability, patient preferences, comorbidities and age, prior adverse events, and quality of life considerations.

An individualized approach to managing cTTP considers several key factors to optimize treatment (Figure 2) [1,6,7,18]. These include the patient’s residual ADAMTS-13 levels, the availability of treatment products such as rADAMTS-13 and plasma infusions, and patient preferences regarding treatment frequency and administration [6,7]. Comorbidities and age are also important factors to consider, as these may require adjustments to the management plan, especially in pediatric or elderly patients [11].

QoL considerations are central, with a focus on minimizing the treatment burden and maximizing well-being. Additionally, a history of adverse events from prior therapies should be reviewed to avoid complications and ensure safer, more effective treatment [6]. This personalized approach balances efficacy, safety, and patient-centered care.

The care team managing cTTP typically includes a range of specialists such as hematologists, nephrologists, neurologists, cardiologists, obstetricians, nurses, social workers, and pharmacists [18,30]. This multidisciplinary team plays a crucial role in the rapid assessment and diagnosis of acute TTP episodes, coordinating emergency treatments and long-term monitoring of ADAMTS-13 levels. Additionally, they manage therapies, address potential organ complications, and provide patient education and support. Effective communication across disciplines is essential to ensure comprehensive and coordinated care for cTTP patients (Supplementary Video 3).

## 2 Discussion

cTTP is increasingly being recognized not only for acute episodes but also for its long-term complications, including stroke, cardiac disease, and renal impairment [1,20]. These findings raise important considerations about the need for ongoing monitoring of silent organ damage, even in clinically stable patients [6,12,13]. While plasma therapy remains the cornerstone of prophylaxis, it is associated with challenges, including volume overload, allergic reactions, and the need for reliable venous access [19,20,26,32]. Certain patient populations may be particularly vulnerable to these complications, highlighting a need for individualized risk assessment.

The development of rADAMTS-13 offers a targeted enzyme replacement strategy with potential advantages over plasma-based therapy [27]. As rADAMTS-13 becomes more widely available, considerations around its cost, accessibility, and integration into standard practice are increasingly relevant. Management during pregnancy presents unique challenges, requiring tailored prophylactic approaches and multidisciplinary coordination to mitigate maternal and fetal risks.

Research is ongoing to better define optimal dosing strategies and long-term safety profile of rADAMTS-13, particularly across different patient subgroups, such as those with renal or cardiovascular comorbidities. There is also growing interest in identifying biomarkers that can more accurately predict disease activity and risk of organ damage, supporting a move toward more personalized monitoring protocols. Emerging tools, such as disease-specific QoL measures, are shifting the focus of cTTP management toward broader outcomes beyond acute event prevention.

These developments prompt ongoing discussions regarding standardized surveillance protocols, the appropriate use of prophylactic therapies, and strategies to optimize long-term patient outcomes. Advances in therapeutic options may ultimately redefine the natural history of cTTP.

### 3 Conclusion

cTTP is a rare, life-threatening disorder caused by inherited ADAMTS-13 deficiency, leading to recurrent clots and organ damage. Plasma infusions are standard but burdensome and carry risks for patients. rADAMTS-13 offers a safer, more targeted, and potentially more effective alternative for long-term prevention and acute management.

## Supplementary material

Supplementary Material 2

Supplementary Video 1 Practitioners and researchers must always rely on their own experience and knowledge in evaluating and using any information, methods, compounds or experiments described herein. Because of rapid advances in the medical sciences, in particular, independent verification of diagnoses and drug dosages should be made. To the fullest extent of the law, no responsibility is assumed by the publisher for any injury and/or damage to persons or property as a matter of products liability, negligence or otherwise, or from any use or operation of any methods, products, instructions or ideas contained in the material herein.

Supplementary Video 2 Practitioners and researchers must always rely on their own experience and knowledge in evaluating and using any information, methods, compounds or experiments described herein. Because of rapid advances in the medical sciences, in particular, independent verification of diagnoses and drug dosages should be made. To the fullest extent of the law, no responsibility is assumed by the publisher for any injury and/or damage to persons or property as a matter of products liability, negligence or otherwise, or from any use or operation of any methods, products, instructions or ideas contained in the material herein.

Supplementary Video 3 Practitioners and researchers must always rely on their own experience and knowledge in evaluating and using any information, methods, compounds or experiments described herein. Because of rapid advances in the medical sciences, in particular, independent verification of diagnoses and drug dosages should be made. To the fullest extent of the law, no responsibility is assumed by the publisher for any injury and/or damage to persons or property as a matter of products liability, negligence or otherwise, or from any use or operation of any methods, products, instructions or ideas contained in the material herein.
