# Supplementary material for: Optimizing the management of congenital thrombotic thrombocytopenic purpura
Source: Res Pract Thromb Haemost. 2026 Jan 20;9(Suppl 4):103270. doi: 10.1016/j.rpth.2025.103270 (PMC12866082; doi:10.1016/j.rpth.2025.103270)
Supplement: Supplementary Figure [file mmc1.pptx]

## Slide 1
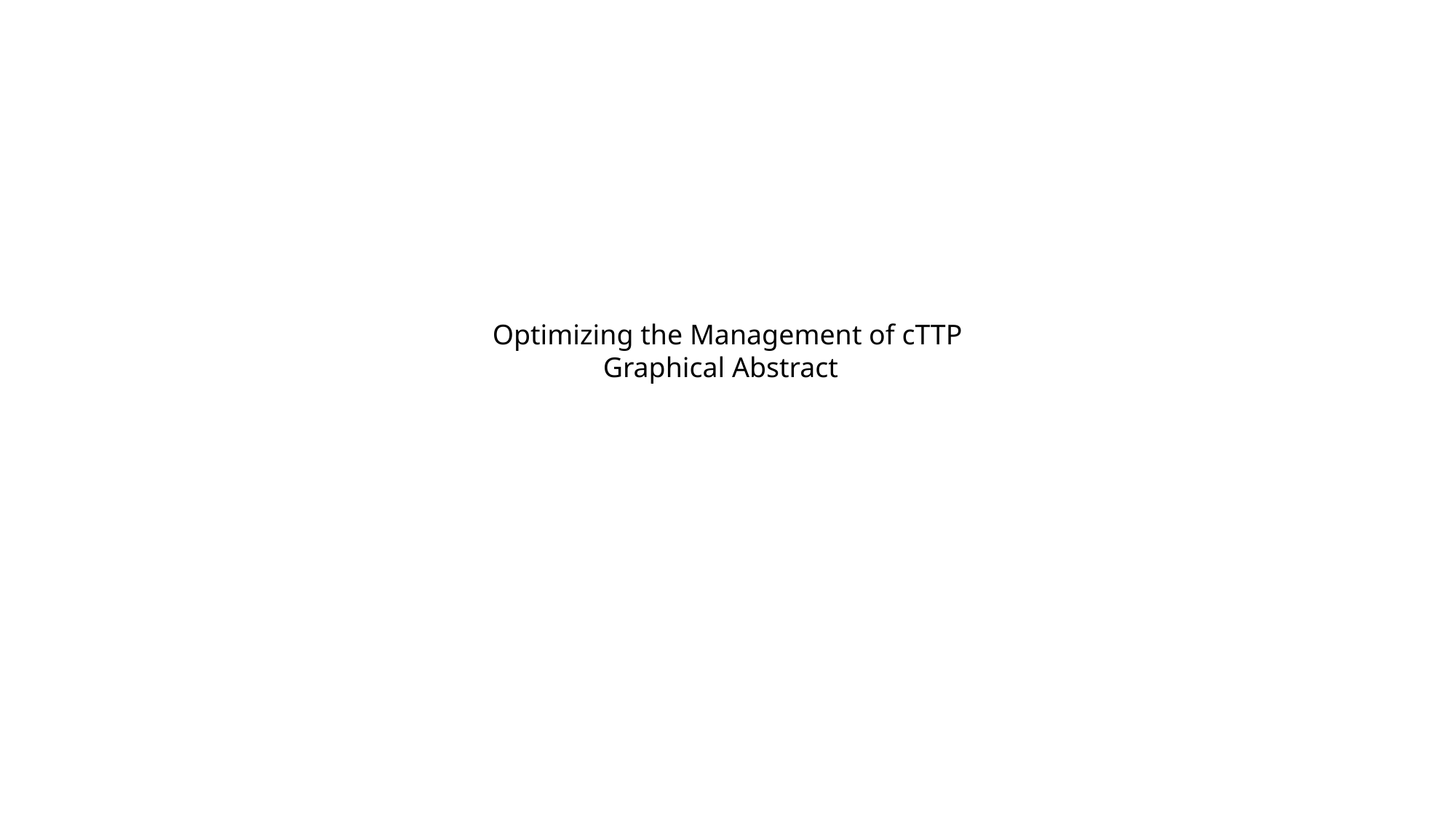

Optimizing the Management of cTTP
Graphical Abstract

## Slide 2
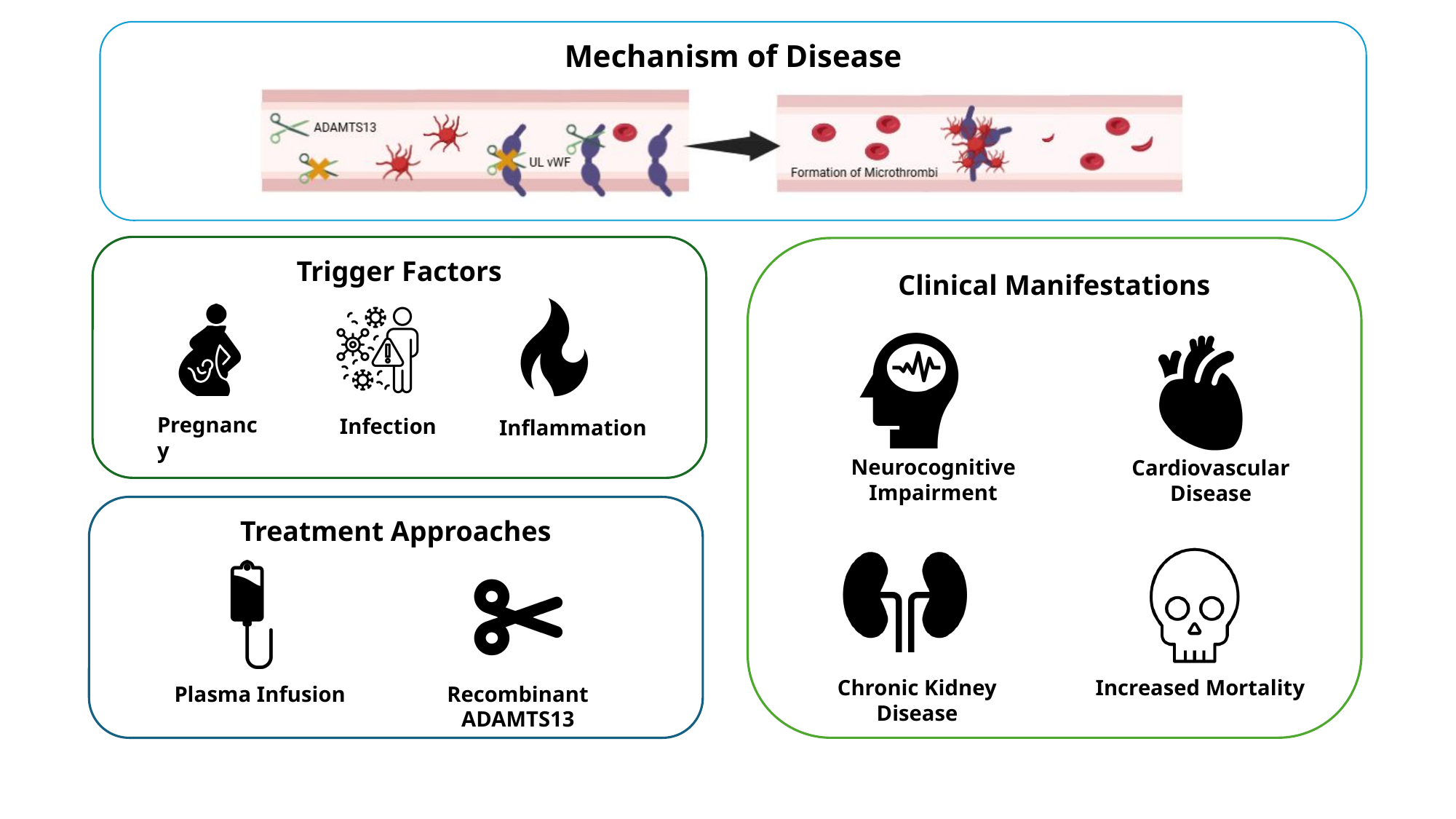

Mechanism of Disease
Trigger Factors
Clinical Manifestations
Pregnancy
Infection
Inflammation
Neurocognitive Impairment
Cardiovascular Disease
Treatment Approaches
Chronic Kidney Disease
Increased Mortality
Recombinant ADAMTS13
Plasma Infusion
