# Supplementary material for: Optimizing the management of congenital thrombotic thrombocytopenic purpura
Source: Res Pract Thromb Haemost. 2026 Jan 20;9(Suppl 4):103270. doi: 10.1016/j.rpth.2025.103270 (PMC12866082; doi:10.1016/j.rpth.2025.103270)
Supplement: Supplementary Material 1 [file mmc2.docx]

| Activity Title | Optimizing the Management of cTTP |
| --- | --- |
| Topic | **cTTP** |
| Accreditation Type | **Physicians**—maximum of 1.0*AMA PRA Category 1 Credit(s)*™ |
| Accreditor | The France Foundation |
| Release Date | July 31, 2025 |
| Expiration Date | July 30, 2026 |
| Estimated Time to Complete Activity | **1.0 hour** |

**TARGET AUDIENCE**

This educational activity is intended for all members of hematology care teams, including physicians, nurse practitioners, and PAs.

**PROGRAM OVERVIEW**

The aim of this educational program is to enhance clinicians' knowledge and confidence about cTTP, addressing the unmet needs of patients with this rare disorder.

**Learning Objectives**

- Recognize common issues faced by patients diagnosed with cTTP, including the high burden of disease during acute episodes, manifestations during sub-acute episodes, and long-term organ damage
- Evaluate benefits and limitations of current treatment approaches for patients with cTTP
- Select prophylactic cTTP treatment approaches to prevent sub-acute manifestations and long-term organ damage

**Faculty**

**Corresponding Author:**

**Melissa F. Glasner, MSc**

Manager, Medical Content

The France Foundation

Old Lyme, Connecticut

United States of America

**Lead Author:**

**Senthil Sukumar, MD**

Assistant Professor, Hematology & Oncology

Baylor College of Medicine

Houston, Texas

USA

**Spero R. Cataland, MD**

Professor of Internal Medicine, Division of Hematology

The Arthur G. James Cancer Hospital and Richard J. Solove Research Institute

Ohio State University

Columbus, Ohio

USA

**Marie Scully, MD**

Professor of Hemostasis and Thrombosis

University College London

Clinical Lead

Hematopathology and Blood Transfusion

University College London Hospitals

London, England

UK

**Shruti Chaturvedi, MBBS, MSCI**

Associate Professor of Medicine

Division of Hematology

Johns Hopkins University

Baltimore, Maryland

USA

**PLANNING COMMITTEE**

**Senthil Sukumar, MD**

Assistant Professor, Hematology & Oncology

Baylor College of Medicine

Houston, Texas

USA

**Spero R. Cataland, MD**

Professor of Internal Medicine, Division of Hematology

The Arthur G. James Cancer Hospital and Richard J. Solove Research Institute

Ohio State University

Columbus, Ohio

USA

**Marie Scully, MD**

Professor of Hemostasis and Thrombosis

University College London

Clinical Lead

Hematopathology and Blood Transfusion

University College London Hospitals

London, England

UK

**Shruti Chaturvedi, MBBS, MSCI**

Associate Professor of Medicine

Division of Hematology

Johns Hopkins University

Baltimore, Maryland

USA

**Senthil Sukumar, MD**

Assistant Professor, Hematology & Oncology

Baylor College of Medicine

Houston, Texas

USA

## INSTRUCTIONS FOR PARTICIPATION AND REQUESTING CREDIT

There are no fees for participating and receiving credit for this activity. Successful completion is achieved by reviewing the continuing education information, completing the activity, and completing the posttest and evaluation form.

**Credit Fulfillment**

- If you are requesting AMA credits or a certificate of participation, your certificate will be available for download

**ACCREDITED PROVIDER**

This activity is jointly provided by The France Foundation and the International Society on Thrombosis and Haemostasis.


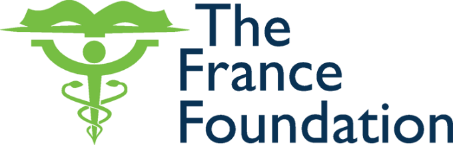

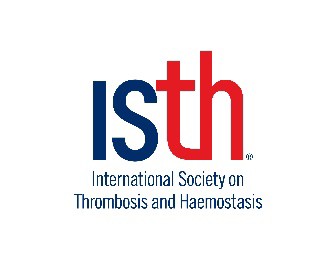


**Accreditation Statement**


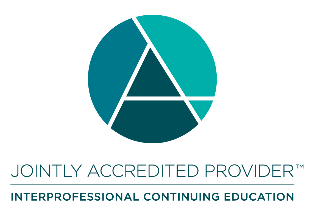
In support of improving patient care, this activity has been planned and implemented by The France Foundation (TFF) and the International Society on Thrombosis and Haemostasis (ISTH). The France Foundation is jointly accredited by the Accreditation Council for Continuing Medical Education (ACCME), the Accreditation Council for Pharmacy Education (ACPE), and the American Nurses Credentialing Center (ANCC) to provide continuing education for the health care team.

## Physician Credit Designation


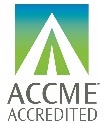
The France Foundation designates this enduring activity for a maximum of 1.0 AMA PRA Category 1 Credit(s)™. Physicians should claim only the credit commensurate with the extent of their participation in the activity.

All other health care professionals completing this course will be issued a statement of participation.

**DISCLOSURE POLICY**

In accordance with the ACCME Standards for Integrity and Independence in Accredited Continuing Education, The France Foundation (TFF) and International Society on Thrombosis and Haemostasis (ISTH) require that individuals in a position to control the content of an educational activity disclose all relevant financial relationships with any commercial entity. TFF and ISTH resolve all conflicts of interest to ensure independence, objectivity, balance, and scientific rigor in all their educational programs.

Furthermore, TFF and ISTH seek to verify that all scientific research referred to, reported, or used in a CME/CE activity conforms to the generally accepted standards of experimental design, data collection, and analysis. TFF and ISTH are committed to providing learners with high-quality CME/CE activities that promote improvements in health care and not those of a commercial interest.

Planning committee members, faculty, reviewers, and activity staff have disclosed the following relevant financial relationships. All relevant financial relationships listed have been mitigated.

| Name of Individual | Role in Activity | Name of Commercial Interest(s) | Nature of Relationship(s) | Mechanism(s) implemented to resolve conflict of interest |
| --- | --- | --- | --- | --- |
| Spero R. Cataland, MD | Planning Committee Member, Faculty | 1. Sanofi, Takeda, Novartis 2. GC Biopharma (S Korea) | 1. Non-Continuing Education Consulting 2. Contract Research | All final planning decisions concerning content, learning objectives, and evaluation questions were made by the non-conflicted planning committee member  Content was reviewed by a non-conflicted content reviewer to ensure that it is not commercially biased, is fair-balanced, and is based on scientific evidence and/or clinical reasoning |
| Shruti Chaturvedi, MBBS | Planning Committee Member, Faculty | 1. Alexion, BioCryst, Kyowa Kirin, Novartis, Sanofi, Sobi, Takeda | 1. Non-Continuing Education Consulting | All final planning decisions concerning content, learning objectives, and evaluation questions were made by the non-conflicted planning committee member  Content was reviewed by a non-conflicted content reviewer to ensure that it is not commercially biased, is fair-balanced, and is based on scientific evidence and/or clinical reasoning |
| Marie Scully, MD | Planning Committee Member, Faculty | 1. Takeda 2. Alexion, Octapharma, Sanofi, Takeda 3. Alexion, Takeda | 1. Non-Continuing Education Consulting 2. Non-Continuing Education Speakers Bureau 3. Contract Research | All final planning decisions concerning content, learning objectives, and evaluation questions were made by the non-conflicted planning committee member  Content was reviewed by a non-conflicted content reviewer to ensure that it is not commercially biased, is fair-balanced, and is based on scientific evidence and/or clinical reasoning |
| Senthil Sukumar, MD | Planning Committee Member, Faculty | 1. Sanofi-Genzyme | 1. Non-Continuing Education Consulting | All final planning decisions concerning content, learning objectives, and evaluation questions were made by the non-conflicted planning committee member  Content was reviewed by a non-conflicted content reviewer to ensure that it is not commercially biased, is fair-balanced, and is based on scientific evidence and/or clinical reasoning |
| Amanda Noe | TFF Staff | No relevant financial relationships | NA | NA |
| Cary Clark | ISTH Staff | No relevant financial relationships | NA | NA |
| Nathan Koenning | ISTH Staff | No relevant financial relationships | NA | NA |
| Heather Tarbox, MPH | TFF Staff | No relevant financial relationships | NA | NA |
| Patrick Harty, PhD | Planning Committee Member, TFF Content Reviewer | No relevant financial relationships | NA | NA |
| Melissa Glasner, MS | Planning Committee Member, TFF Content Reviewer | No relevant financial relationships | NA | NA |
| Miriam Giles | TFF Staff | No relevant financial relationships | NA | NA |

**Disclosure of Unlabeled Use:**

TFF and ISTH require CME faculty (speakers) to disclose when products or procedures being discussed are off label, unlabeled, experimental and/or investigational and any limitations on the information that is presented, such as data that are preliminary or that represent ongoing research, interim analyses, and/or unsupported opinion. Faculty in this activity may discuss information about pharmaceutical agents that are outside of US Food and Drug Administration approved labeling.

This information is intended solely for continuing medical education and is not intended to promote off-label use of these medications. TFF and ISTH do not recommend the use of any agent outside of the labeled indications. If you have questions, contact the Medical Affairs Department of the manufacturer for the most recent prescribing information.

**COMMERCIAL SUPPORT ACKNOWLEDGMENT**

This activity is supported by an independent educational grant from Takeda.

**Disclaimer**

The France Foundation and ISTH present this information for educational purposes only. The content is provided solely by faculty who have been selected because of recognized expertise in their field. Participants have the professional responsibility to ensure that products are prescribed and used appropriately on the basis of their own clinical judgment and accepted standards of care. The France Foundation, ISTH, and the commercial supporter(s) assume no liability for the information herein.

**CONTACT INFORMATION**

**If you have questions about this CME activity, please contact The France Foundation at 860-434-1650 or** [info@francefoundation.com](mailto:info@francefoundation.com)**.**

**COPYRIGHT INFORMATION**
Copyright © 2025 The France Foundation. Any unauthorized use of any materials on the site may violate copyright, trademark, and other laws. You may view, copy, and download information or software ("materials") found on the site subject to the following terms, conditions, and exceptions:

- The materials are to be used solely for personal, noncommercial, informational, and educational purposes. The materials are not to be modified. They are to be distributed in the format provided with the source clearly identified. The copyright information or other proprietary notices may not be removed, changed, or altered.
- Materials may not be published, uploaded, posted, or transmitted (other than as set forth herein) without The France Foundation's prior written permission

**PRIVACY POLICY**
The France Foundation protects the privacy of personal and other information regarding participants and educational collaborators. The France Foundation will not release personally identifiable information to a third party without the individual's consent, except when such information is required for reporting purposes to the ACCME.

The France Foundation maintains physical, electronic, and procedural safeguards that comply with federal regulations to protect against the loss, misuse, or alteration of information that we have collected from you.

Additional information regarding The France Foundation’s Privacy Policy can be viewed at <http://www.francefoundation.com/privacy>.

**CONTACT INFORMATION**

**If you have questions about this CME activity, please contact The France Foundation at 860-434-1650 or** [info@francefoundation.com](mailto:info@francefoundation.com)**.**

[CLICK](http://comp.realcme.com/cms/user?TB_iframe=true&width=655) **HERE TO BEGIN ACTIVITY**

**Please review this information, and proceed below**
