# Supplementary material for: Optimizing the management of congenital thrombotic thrombocytopenic purpura
Source: Res Pract Thromb Haemost. 2026 Jan 20;9(Suppl 4):103270. doi: 10.1016/j.rpth.2025.103270 (PMC12866082; doi:10.1016/j.rpth.2025.103270)
Supplement: Supplementary Material 2 [file mmc3.docx]

Thank you for recently participating in the activity titled “*Optimizing the Management of cTTP*.” To help us assess the effectiveness of the program, please spend a few moments answering the questions below. Upon acceptance of this form by The France Foundation, you will receive, via email, a certificate documenting your successful completion of this activity.

## DEMOGRAPHICS

1. Health Care Professional Category

Physician

Pharmacist

PA

APRN

RN

Other: TEXT BOX

1. Area of Primary Clinical Focus

Hematology

OB/GYN

Primary Care

Pediatrics

Family Medicine

Other: TEXT BOX

1. What age group are most of your patients in?

Pediatric (Birth-12 years)

Adolescent (13-18 years)

Adult (19-64 years)

Geriatrics (65+ years)

1. Number of years in practice

0

1-5

6-10

11-15

16-20

21-25

> 25

N/A, not a clinician

1. How many of your patients are being managed/cared for with cTTP each month?

0

1-5

6-10

11-15

16-20

> 20

N/A, not involved in patient care

**---------------------------------------------------------------------------------------------------------------------**

## POST-TEST

## Which of the following is a potential long-term complication of recurrent cTTP episodes?

## Lung fibrosis

## Recurrent strokes

## Liver cirrhosis

## Bone marrow failure

## Rationale: Recurrent episodes of cTTP can cause ongoing microvascular thrombosis, which significantly increases the risk of long-term complications such as recurrent strokes.

## How might rADAMTS13 therapy impact the quality of life for cTTP patients?

## By increasing the rate of plasma transfusion

## By allowing for oral administration at home

## By curing the underlying genetic defect

## By reducing the frequency of hospital visits

## Rationale: rADAMTS13 therapy can decrease the need for frequent plasma infusions and emergent treatments, thereby significantly reducing the frequency of hospital visits for cTTP patients.

## How can a health care team best manage the trade-offs between treatment efficacy and side effects in cTTP patients?

## By including psychological evaluations in follow-up care

## By regularly assessing and adjusting the treatment based on patient feedback and side effect profile

## By choosing the most effective treatment regardless of side effects

## By referring patients to community organizations for social support

## Rationale: Optimal management of cTTP requires clinicians to regularly evaluate both treatment efficacy and side effects, incorporating patient input to adjust therapy as needed. This individualized approach helps achieve disease control while minimizing negative impacts from treatment, rather than prioritizing efficacy alone or relying only on external support.

## SELF-ASSESSMENT

1. Please rate your skills/strategy to apply the following in your practice BEFORE and AFTER this activity:

|  | NA | 1 low | 2 | 3 | 4 | 5 high |
| --- | --- | --- | --- | --- | --- | --- |
| Recognize common issues faced by patients diagnosed with cTTP, including the high burden of disease during acute episodes, manifestations during sub-acute episodes, and long-term organ damage |  | BEFORE |  |  |  |  |
|  |  | AFTER |  |  |  |  |
| Evaluate the benefits and limitations of current treatment approaches for patients with cTTP |  | BEFORE |  |  |  |  |
|  |  | AFTER |  |  |  |  |
| Select prophylactic cTTP treatment approaches in order to prevent sub-acute manifestations and long-term organ damage |  |  | BEFORE |  |  |  |
|  |  |  | AFTER |  |  |  |

## PRACTICAL APPLICATION

1. Do you intend to apply new skills/strategies to your practice as a result of this activity?

Yes*  I’m possibly considering changes*

This activity affirms what I already do in practice  No, I do not intend to make any changes†

*If yes or possibly considering changes, please check off what you intend to do differently or incorporate into your clinical management of patients as a result of this educational activity.

I will closely monitor patients for manifestations of sub-acute episodes of cTTP and address any potential symptoms

I will stay up to date on the improved benefits and/or reduced limitations of new cTTP therapies compared to current standards of care

I will tailor prophylactic treatment plans based on individual patient risk factors to prevent both sub-acute symptoms and long-term complications

I will regularly assess treatment efficacy and adjust prophylactic strategies to ensure the best outcomes for long-term organ health and cTTP management

Other, please specify______________________________________________________________________

†If you do not intend to make any changes, please explain why not: ___________________________________________

1. Did this activity provide knowledge or skills/strategies that fulfilled your educational need?  Yes  No
2. Please provide a specific way in which this activity will impact your patient care. TEXT BOX
3. Did this activity demonstrate ways to overcome barriers to applying new skills/strategies in your practice, particularly related to your role on the health care team?

Yes

If yes, how: __________________

No

1. When discussing prophylaxis with patients, what barriers have you experienced related to their understanding and acceptance of the benefits of this new therapy?

## SATISFACTION

1. Was the format of this educational activity appropriate for the content presented?

Yes

No

1. How might the format of this activity be improved? TEXT BOX
2. Which of the following educational topics related to cTTP would you like to see addressed in future activities? (select all that apply)

ISTH cTTP treatment guidelines

Discussing prophylaxis with patients

Long-term management of cTTP

Considerations for special populations with cTTP

Other: TEXT BOX

1. Was this activity scientifically sound and free of commercial bias or influence?

Yes

No

If no, please explain: TEXT BOX

**---------------------------------------------------------------------------------**

1. The France Foundation would like to send you educational opportunities relevant to your practice, emailed to the address you provided. May we have your consent?

Yes

No
